# Metabolic Profiles of Feline Obesity Revealed by Untargeted and Targeted Mass Spectrometry-Based Metabolomics Approaches

**DOI:** 10.3390/vetsci12080697

**Published:** 2025-07-25

**Authors:** Renata Barić Rafaj, Ivana Rubić, Josipa Kuleš, Dominik Prišćan, Alberto Muñoz-Prieto, Jelena Gotić, Luka Ećimović, Nada Kučer, Marko Samardžija, Mislav Kovačić, Vladimir Mrljak

**Affiliations:** 1 Department of Chemistry and Biochemistry, Faculty of Veterinary Medicine, University of Zagreb, 10000 Zagreb, Croatia; rrafaj@vef.unizg.hr; 2 Laboratory of Proteomics, Clinic for Internal Diseases, Faculty of Veterinary Medicine, University of Zagreb, 10000 Zagreb, Croatia; irubic@vef.unizg.hr (I.R.); dpriscan@vef.unizg.hr (D.P.); 3 Interdisciplinary Laboratory of Clinical Pathology, Interlab-UMU, Campus of Excellence Mare Nostrum, University of Murcia, 30100 Murcia, Spain; alberto.munoz@um.es; 4 Clinic for Internal Diseases, Faculty of Veterinary Medicine, University of Zagreb, 10000 Zagreb, Croatia; jselanec@vef.unizg.hr (J.G.); nkucer@vef.unizg.hr (N.K.); vmrljak@vef.unizg.hr (V.M.); 5 Clinical Pathology Laboratory, University Animal Hospital, Swedish University of Agricultural Sciences, SE-75007 Uppsala, Sweden; lecimovic@vef.unizg.hr; 6 Clinic for Reproduction and Obstetretic, Faculty of Veterinary Medicine, University of Zagreb, 10000 Zagreb, Croatia; smarko@vef.unizg.hr; 7 Department of Biology, J.J. Strossmayer University of Osijek, 31000 Osijek, Croatia

**Keywords:** metabolomics, feline, obesity, mass spectrometry

## Abstract

Obesity is currently one of the major medical problems affecting humans and companion animals, including cats; however, a detailed understanding of the metabolic processes altered in feline obesity remains limited. This study aimed to investigate obesity-related changes in the serum metabolome of three groups of cats, metabolically healthy normal-weight (MHN) cats, metabolically healthy overweight (MHO) cats, and metabolically unhealthy overweight (MUO) cats. Metabolome changes were assessed using LC-MS (untargeted), LC-MS (targeted), and FIA-MS (targeted) methods. Untargeted analysis detected 141 significant annotated features, while targeted approach identified 48 metabolites significantly associated with obesity. Both untargeted and targeted analyses showed lower kynurenine levels in the MUO group compared to the MHN group. Targeted LC-MS analysis identified 11 significant metabolites, whereas the FIA-MS approach detected 37. Four metabolites—glycine, citrulline, and two phosphatidylcholines—were found at lower levels in the MHO group compared to the MHN group. Arginine and proline metabolism, along with methionine metabolism, were significantly altered pathways, while thyroid hormone synthesis was independently altered with the highest enrichment ratio. The obtained results suggest that cats with a healthy phenotype exhibit an intermediate-metabolic-risk profile and provide new insights into the metabolic mechanisms and pathways underlying feline obesity.

## Simple Summary

Today, obesity is a major health problem in pets and people. Although metabolic dysfunction is a common problem in obesity, some individuals remain metabolically healthy and do not show typical signs of obesity-related disorders. The aim of this study was to investigate overweight and obesity-related changes in the serum of metabolically healthy normal-weight cats, metabolically healthy overweight cats, and metabolically unhealthy overweight cats. Reduced glycine could be an early predictor of impaired glucose tolerance and insulin resistance. The major metabolites contributing to obesity were lipid metabolites. Disturbances in thyroid hormone synthesis were also identified. Many of the metabolites found have been previously associated with impaired glucose and energy metabolism and the potential development of insulin resistance. Therefore, it would be valuable to investigate whether these altered metabolites play a role in the etiology of feline-obesity-related metabolic diseases.

## 1. Introduction

Declared as an epidemic by the World Health Organization [1], obesity is today present at pandemic levels in both humans and animals. As a complex disorder, this state is a result of an imbalance between energy expenditure and caloric intake. Associated with increased morbidity and mortality, obesity has been declared as a major threat to pet health by the British Small Animal Veterinary Association [2]. It is estimated that 12–63% of cats that are kept as pets are overweight [3].

Despite the increasing prevalence of overweight and obesity in cats, the metabolic changes related to those conditions are not well understood. To date, the most metabolic studies on the impact of obesity have been focused on humans, with relatively few addressing feline obesity. In a recent study, Pallotto et al. [4] applied a metabolomics approach to examine the effects of weight loss and diet on the serum metabolome of cats, identifying several biomarkers of weight loss. Ohlund et al. [5] concluded that detection of an altered metabolome might identify cats at risk of developing diabetes. Reeve-Johnson [6] investigated metabolic profiles associated with spontaneous obesity in senior cats and identified specific alterations in lipid and amino acid profiles, as well as disturbances in glycolic acid metabolism. Additionally, Gottlieb et al. [7] applied metabolomics to compare plasma metabolites between diabetic cats in remission and healthy controls, finding broad metabolic differences.

It has been shown that metabolically healthy obese humans are still at risk of developing metabolic abnormalities in the future; however, the specific metabolic mechanisms and pathways involved, as well as the progression of changes, remain unclear [8]. To date, metabolically healthy obesity in cats has not been characterized in terms of comprehensive risk assessment or associated metabolite changes compared to normal-weight cats.

This preliminary study investigates feline obesity by evaluating metabolome changes associated with excess weight using both targeted and untargeted metabolomics approaches. The primary aim was to identify specific metabolites altered in overweight cats that may contribute to metabolic dysfunction. A secondary goal was to identify metabolic signatures and potential biomarkers of so-called metabolically healthy obesity. Additionally, this study aimed to uncover the metabolic pathways involved in these changes.

## 2. Materials and Methods

### 2.1. Study Design and Cohorts

A detailed history of all animals was obtained. Cats were with normal weight and overweight or obese for at least 6 months, and considered clinically healthy at the time of examination. Exclusion criteria were acute or chronic disease in the last 2 months, use of medication or a dietary supplement, or a recent change in body weight. Neutered cats were excluded from the study. The cats were crossbreeds, and all the animals were fed with a commercial diet at home by the owners. A scale of 1–9 was used to estimate body condition score (BCS), where 5 indicates normal weight, 6 and 7 overweight, and 8 and 9 obesity [9]. Each animal was assigned a BCS by 2 veterinarians who independently estimated BCS, and the average result was used. According to BCS and laboratory data, cats were divided into 3 groups—metabolically healthy normal-weight cats (MHNs, n = 10), metabolically healthy overweight and obese cats (MHOs, n = 10) and metabolically unhealthy overweight and obese cats (MUOs, n = 8 for targeted, or n = 7 for untargeted metabolomics).

### 2.2. Participants

A total of 28 cats of different breeds were included in the study: the MHN group comprised 5 males and 5 females, aged 4–13 years, with body weights of 4.8–5.6 kg, BCSs of 5 for each animal; the MHO group comprised 5 males and 5 females, aged 5–12 years, with body weights of 5.2–7.0 kg and BCSs of 6–9; and the MUO group comprised 4 males and 4 females, aged 5–10 years, with body weights of 5.5–7.5 kg and BCSs of 6–8 (Supplementary Table S1).

### 2.3. Diagnoses and Classification

Hematological measurements were performed using an automatic analyzer, “Horiba ABX cell counter” (Diagnostics, Montpellier, France). Biochemical analyses (urea, creatinine, total proteins, albumins, total bilirubin, glucose, aspartate aminotransferase, alanine aminotransferase, alkaline phosphatase, triglycerides, cholesterol) were carried out with an automatic analyzer Olympus AU600 (Olympus Corporation, Tokyo, Japan), using standardized methods and original reagents from the manufacturer. Adiponectin was measured by the ELISA test (Biotang, Source International, Camarillo, CA, USA).

Metabolically healthy obesity and metabolically unhealthy obesity represent two distinct phenotypes within the obese population of cats, characterized by the presence or absence of metabolic abnormalities. The hematological and biochemical parameters of metabolically healthy cats in the MHN and MHO groups were all within the normal range. To identify the metabolic disturbances connected with obesity, we used feline metabolic syndrome diagnostic criteria proposed by Okada et al. [10]. The MUO group included cats with a higher BCS, fasting glucose concentration (>6.7 mmol/L), triglycerides (>1.86 mmol/L) or total cholesterol (>4.7 mmol/L), and lower adiponectin (3.0 < μg/mL). The results are shown in Table 1.

### 2.4. Sample Collection

Blood was taken after 8 h of fasting, from the cephalic vein, in two tubes, one with EDTA anticoagulant for hematological tests, and the other with a coagulation activator gel, for biochemical tests. After the clotting process, the serum tube was centrifuged at 1200× *g* for 10 min, after which a portion of the serum was separated for routine biochemical analyses and a portion stored at −80 °C until spectroscopic measurements were performed.

### 2.5. Untargeted and Targeted Metabolomic Analyses

#### 2.5.1. Untargeted Metabolomics

Metabolites for untargeted metabolomics analysis were extracted using a chloroform/methanol/water (1:3:1, v/v/v) mixture (chloroform, methanol (Honeywell, Charlotte, NC, USA), water (Merck, Darmstadt, Germany)) [11,12]. Serum samples (25 µL) were mixed with extraction solvent on a 4 °C vortex mixer for 5 min. Pooled samples were prepared using 10 µL from each serum. All samples were centrifuged at 13,000× *g* for 5 min at 4 °C, and 200 µL of supernatant was stored at −80 °C for UHPLC-MS/MS analysis.

Serum extracts were analyzed using a Dionex UltiMate 3000 UHPLC system coupled with a Thermo Orbitrap Q Exactive Plus MS (Thermo Fisher Scientific, Bremen, Germany). Separation was performed on a ZIC-pHILIC column (150 × 4.6 mm, 5 μm, Merck Sequant, Darmstadt, Germany) via HILIC at 25 °C. To prevent retention time shifts, all samples were run in a single sequence. A linear gradient from 80% to 5% mobile phase B (acetonitrile) over 15 min was used, followed by 2 min at 5% B, a return to 80% B in 1 min, and 6 min equilibration. Mobile phase A was 20 mM ammonium carbonate in water. The flow rate was 0.3 mL/min with 10 μL injections, and samples were kept at 5 °C in the autosampler. MS was operated in positive and negative electrospray modes at 70,000 resolution, scanning *m*/*z* 70–1050. The source voltage was ±3.8 kV, with sheath gas at 40, auxiliary gas at 5 (arbitrary units), and capillary temperature at 320 °C.

Metabolites were identified using a standard mix of ~150 reference compounds (from Glasgow Polyomics, Glasgow, UK) dissolved in acetonitrile (90 μL acetonitrile + 10 μL standard mix). Quality control samples, prepared from beer and human urine, were used to check signal reproducibility and chromatography quality.

Data processing was performed using MSconvert (ProteoWizard Software Foundation, San Diego, CA, USA) and the Polyomics integrated Metabolomics Pipeline (PiMP) at http://polyomics.mvls.gla.ac.uk (accessed on 23 July 2025) [13]. Raw LC-MS data were converted from RAW to mzXML format, centroided, and separated by polarity using ProteoWizard software Version 3 [14]. Metabolites were identified in PiMP software following MSI guidelines. Identification was based on mass and retention time matching with authentic standards, while annotation relied on accurate mass searches in databases such as HMDB (Human Metabolome Database) and KEGG, integrated within PiMP.

#### 2.5.2. Targeted Metabolomics

Metabolite extraction for targeted metabolomics was performed using the Absolute IDQ p400 kit (Biocrates Life Science AG, Innsbruck, Austria). This kit allows analysis of up to 408 metabolites. It uses the liquid chromatography–mass spectrometry (LC-MS/MS) to quantify amino acids and biogenic amines, and flow injection analysis–mass spectrometry (FIA-MS/MS) to quantify acylcarnitines, glycerophospholipids, glycerides, hexoses, cholesterol esters, and sphingolipids. Metabolite extraction was performed using a 96-well plate system following the manufacturer’s instructions in three steps: protein removal, internal standard normalization, and derivatization. Serum samples (10 μL), calibration standards, zero standards (phosphate-buffered saline), and quality control samples (QC 1–3, QC2 in five replicates) were added to the plate containing the internal standard mix. Samples were dried for 30 min using a vacuum manifold (Thermo Scientific, Waltham, MA, USA), derivatized with 50 μL of 5% phenylisothiocyanate (PITC) solution (Sigma-Aldrich, St. Louis, MO, USA) in water:ethanol:pyridine (1:1:1; water: Merck, Darmstadt, Germany; ethanol: Honeywell, Charlotte, NC, USA; pyridine: BDH PROLABO, Lutterworth, UK), and incubated for 20 min at room temperature. Plates were dried again for 60 min using the same vacuum manifold. For FIA-MS/MS, metabolites were extracted with 300 μL of 5 mM ammonium acetate (Sigma-Aldrich, St. Louis, MO, USA) in methanol (Honeywell, Charlotte, NC, USA), and collected using the vacuum manifold (Thermo Scientific, Waltham, MA, USA). For LC-MS/MS, 150 μL of extract from the capture plate was transferred and mixed with 150 μL of LC-MS-grade water. Feline serum samples were analyzed on a Dionex Ultimate 3000 UHPLC system (Thermo Fisher Scientific, Germering, Germany) coupled with a Q Exactive Plus Orbitrap mass spectrometer (Thermo Fisher Scientific, Bremen, Germany). Metabolites were separated on a Thermo p400 HR UHPLC column (Biocrates) at 50 °C. Mobile phase A was 0.2% formic acid (Sigma-Aldrich, St. Louis, MO, USA) in water (Merck, Darmstadt, Germany), and mobile phase B was 0.2% formic acid in acetonitrile (Honeywell, Charlotte, NC, USA). A 5 μL injection was used, with a total run time of 5.81 min and a gradient from 0% to 95% B over 4 min at 0.8 mL/min. For FIA-MS/MS, the flow rate changed from 0.05 mL/min (0–1.6 min) to 0.2 mL/min (1.6–2.8 min), and then back to 0.05 mL/min, using the FIA mobile phase. Analyses were performed in both positive and negative electrospray modes according to Biocrates protocols. The mass spectrometer operated in full-scan mode at 70,000 resolution, scanning *m*/*z* 100–800 (LC-MS/MS) and 100–1000 (FIA-MS/MS), with 1 microscan, AGC target of 1 × 10^6^, and 250 ms maximum injection time. HESI source parameters were 3.0 kV source voltage, 300 °C capillary temperature, sheath gas 60, auxiliary gas 30, S-lens RF level 60 (LC1) and 90 (LC2), and aux gas heater at 550 °C for LC-MS/MS; and 2.5 kV source voltage, 300 °C capillary temperature, sheath gas 15, auxiliary gas 5, S-lens RF level 60, and aux gas heater at 120 °C for FIA-MS/MS.

The targeted data analysis was performed using the Biocrates MetIDQ software (Biocrates Life Science AG, Innsbruck, Austria). LC-MS metabolite quantification was performed via XCalibur Quan 4.1 software (Thermo Fisher Scientific, Waltham, MA, USA) based on a 7-point calibration curve and isotope-labeled internal standards for the most analytes. The FIA-MS/MS analysis used a single-point calibrator with representative internal standards.

### 2.6. Statistical Analysis

Statistical analysis of the metabolomics data was performed using the online available data processing platform MetaboAnalyst v.5.0 by means of univariate and multivariate statistical approaches [15]. For untargeted metabolomics, statistical analyses were conducted on the combined positive- and negative-ion datasets, exported from PiMP. We used appropriate parameters for normalization to obtain a normal distribution (Gaussian distribution). Missing values (0.6%) were replaced by 1/5 of the minimum positive value for each variable. The untargeted data were normalized to a constant sum, log-transformed, and Pareto-scaled. In the targeted metabolomics, missing values (4.5%) were replaced by the estimated missing value using KNN (feature-wise). The targeted data were normalized to sample median, log-transformed, and auto-scaled. The overall differences in the metabolic profiles of the three groups of cats were analyzed by the one-way analysis of variance (ANOVA) and Fisher’s least-significant difference (Fisher’s LSD) post hoc test, partial least square–discriminant analysis (PLS-DA), and variable importance on projection (VIP). Metabolites with a *p*-value of <0.05 were considered statistically significant. Correlation between selected markers and phenotype (body weight and BCS) were calculated by Pearson’s rank correlation coefficient.

Metabolite set enrichment analysis was performed using the joint significant metabolites identified by the untargeted and targeted metabolomics approaches using MetaboAnalyst v.5.0. [15]. Metabolite sets (SMPDB; 99 metabolite sets based on normal human metabolic pathways) were used as a metabolite set library in the enrichment analysis.

### 2.7. Ethical Approvals

With approval from the Ethics Committee of the Faculty of Veterinary Medicine, University of Zagreb, systematic health examinations were conducted on pet cats. All owners provided written informed consent before entering the study.

## 3. Results

### 3.1. Data Processing

Using a targeted metabolomics approach, we identified 48 significant metabolites with differential abundance in three groups of cats. Among them, a total of 11 significant metabolites were identified by LC-MS analysis (Table 2).

Using a targeted FIA-MS approach, we detected 37 significant metabolites divided into groups of lysophosphatidylcholines (2) (LPCs), phosphatidylcholines (16) (PCs), triglycerides (19) (TGs), sphingomyelins (2) (SMs), and ceramides (1) (CERs). Four metabolites, glycine (GLY), citruline (CIT), PC (39:5), and PC (42:7), were significantly lower in the MHO group compared with the MHN group. The majority of changes were observed in the MUO group. When comparing the MUO group with the MHN group, 25 metabolite levels were higher, and 19 were lower. All of the metabolites with higher levels were different TGs. All of the measured subspecies of TGs were also higher in the MUO group compared to the MHO group, while kynurenine (KYN), GLY, SM (40:4), PC (42:3), serine (SER), SM (31:1), proline (PRO), PC (33:0), PC (40:7), PC (37:5), LPC (18:1), PC (34:5), CER (34:0), PC (24:0), creatinine (CRE), tryptophane (TRY), LPC (18:2), simmetric dimethylarginine (SDMA), PC-0 (26:1), PC (37:7), PC (44:10), PC (36:5), and putrescine (PUT) were significantly lower (Table 2).

An untargeted metabolomics approach resulted in the detection of 141 significant metabolic features (*p*-value of <0.05). Of these 141 features, only 1, KYN, was confidently identified using retention time (RT) and an accurate mass-to-charge ratio (*m*/*z*), corresponding to Level 1 identification based on the Metabolomics Standards Initiative (MSI) criteria. The remaining features were annotated using *m*/*z* alone, categorized as Level 3 MSI. To ensure the robustness and reliability of our findings, we focused exclusively on metabolites identified at Level 1 MSI.

### 3.2. Discriminant Metabolite Identification

**Figure 1:**
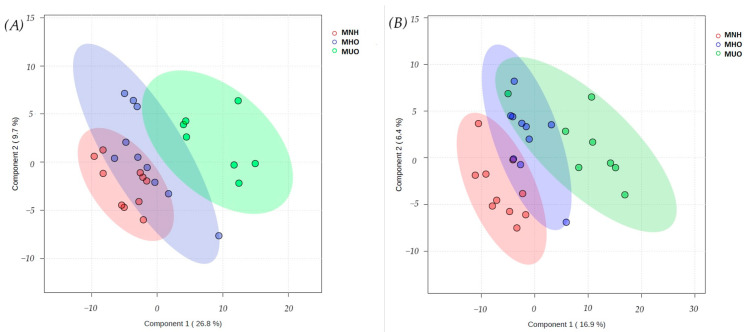
Partial least squares–discriminant analysis (PLS-DA) 3D score plot of serum samples from three groups of cats (MHN (red); MHO (blue); MUO (green) performed by targeted (**A**) and untargeted (**B**) metabolomics analysis, based on 199 identified metabolites (targeted) and 2907 detected peaks (untargeted). MHNs—metabolically healthy normal-weight cats, MHOs—metabolically healthy overweight and obese cats, MUOs—metabolically unhealthy overweight and obese cats.

In order to allow visualization of the data based on disease classification between three groups of cats, PLS-DA was performed. This analysis represented fairly clear intergroup separation between the three experimental groups of cats investigated by targeted and untargeted metabolomics (Figure 1).

The validity of the PLS-DA model was confirmed through cross-validation, which identified the optimal model with four components for targeted metabolomics (R^2^ = 0.95, Q^2^ = 0.56). However, permutation testing (*p* = 0.32, n = 1000) indicated that the classification performance may not be statistically significant, warranting cautious interpretation of the model’s discriminative ability. For untargeted metabolomics, the best classifier model comprised one component (R^2^ = 0.63, Q^2^ = 0.43), indicating a good model fit and acceptable predictive power. Meanwhile, the permutation test produced a *p*-value of 0.065—slightly above the conventional significance threshold (*p* < 0.05)—suggesting that the model may capture meaningful group separation, though the risk of overfitting cannot be entirely excluded. These findings support the use of the model for identifying potential discriminative metabolites, but further validation is warranted.

**Figure 2:**
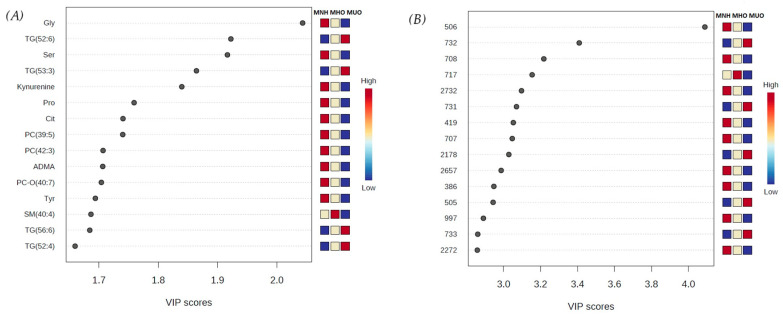
Variable importance in projection (VIP) scores for the 15 most influential metabolites identified by PLS-DA analysis in targeted (**A**) and untargeted (**B**) metabolomics. The intensity of the colored boxed on the right represents the relative concentrations or intensities of the corresponding metabolite in three different groups of cats, with red corresponding to higher concentration/intensity, and blue corresponding to lower concentration/intensity. MHNs—metabolically healthy normal-weight cats, MHOs—metabolically healthy overweight and obese cats, MUOs—metabolically unhealthy overweight and obese cats.

The overall variable importance in projection (VIP) scores from the PLS-DA identified the 15 most highlighted metabolites/features contributing to group separation (Figure 2). For the targeted metabolomics analysis, the results showed that the highest VIP score belonged to GLY. The most important metabolites identified by the targeted metabolomics approach were serine, two TGs (TG (52:6) and (53:3)), and kynurenine. The TGs were higher in the MUO group in comparison to the control, while the concentrations of GLY, SER, and kynurenine were lower. In the untargeted metabolomics, the top five most influential metabolic features obtained by VIP were features 506, 732, 708, 717, and 2732. Among them, feature 506 had the highest VIP score. Feature 732 was higher in the MUO group in comparison to the control group, while features 506, 708, 717, and 2732 were lower (Figure 2).

**Figure 3:**
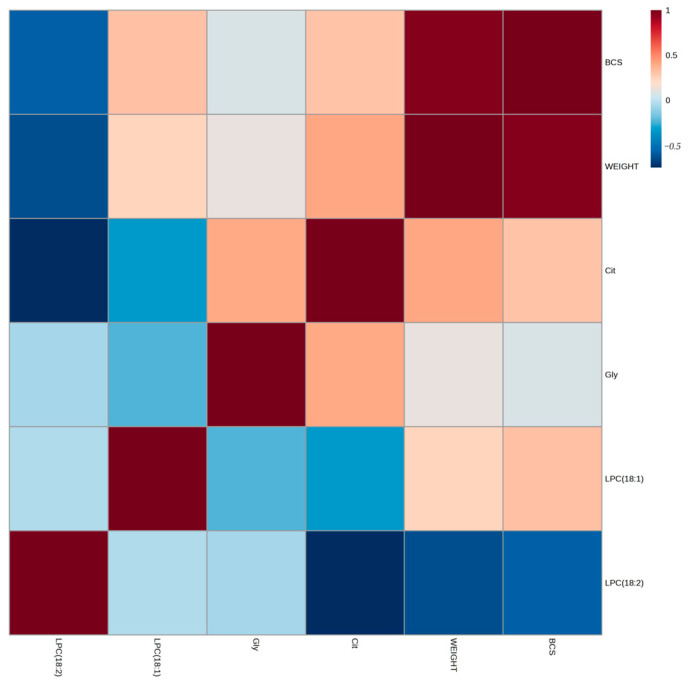
Correlation heatmap for four selected markers (glycine, citrulline, LPC18:1, and LPC18:2) and phenotype parameters (weight and BCS). Correlations were calculated by Pearson’s rank correlation coefficient.

Correlations between four selected markers (glycine, citrulline, LPC18:1, and LPC18:2) and phenotype parameters such as weight and BCS are shown in Figure 3. A moderate association was found between citrulline concentration and the weight of cats (r = 0.41, *p* = 0.03), and a strong negative association was found between LPC18:2 and weight (r = −0.64, *p* < 0.001), as well as between LPC18:2 and BCS (r = −0.58, *p* = 0.001).

### 3.3. Enrichment Analysis

**Figure 4:**
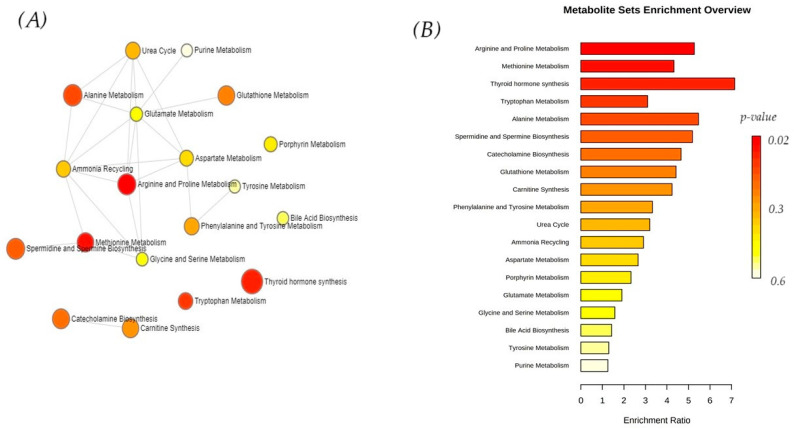
Pathway enrichment displayed as a network view (**A**) and as a bar chart view (**B**) of the significant metabolites identified by untargeted and targeted approaches derived from the metabolic datasets for prediction of pathways associated with serum metabolite sets. Metabolite sets (SMPDB; 99 metabolite sets based on normal human metabolic pathways) were used as a metabolite set library in the enrichment analysis. The colors and the bar length represent the metabolites with different levels of significance according to enrichment analysis.

For the enrichment analysis, joint significant metabolites (*p*-value < 0.05) identified by untargeted and targeted metabolomics were used (peak intensities for metabolites identified by untargeted metabolomics and concentrations for metabolites identified by targeted metabolomics), resulting in 19 pathways which were designated as enriched, as displayed in Figure 4. Arginine, proline metabolism, and methionine metabolism were statistically significant. The most interconnected metabolic pathways were arginine and proline metabolism, glutamate metabolism, and aspartate metabolism, while thyroid hormone synthesis was independently altered. This analysis demonstrated key metabolites participating in arginine and proline metabolism, methionine metabolism, and thyroid hormone synthesis (Figure 4).

## 4. Discussion

Using comprehensive metabolic profiling, we detected 141 significant annotated features with the untargeted approach and 48 metabolites with the targeted method. The majority of metabolites identified in the targeted analysis distinguished the MUO group from the MHN group. Four metabolites were significantly altered in the MHO group compared to the MHN group.

A key finding of this study was the observed link between serum glycine (GLY) concentrations and obesity in both the MHO and MUO groups, where glycine levels were lower compared to the MHN group. Additionally, serine (SER) and proline (PRO) concentrations were lower in the MUO group compared to the MHN group. These results align with previous studies reporting altered circulating amino acid levels in obese animals and humans. In a recent study examining the effects of weight loss on the feline metabolome, glycine and serine levels were found to increase consistently during the weight loss process [4]. Most studies investigating obesity-related metabolic changes have been conducted in humans, where inverse correlations between plasma glycine concentrations and body mass index (BMI) have been well established [16,17,18,19,20]. In addition to metabolically healthy obesity, in metabolic disorders associated with obesity, lower GLY concentrations have also been consistently observed [21,22,23,24]. Palmer et al. [24] emphasized that reduced plasma GLY could be an early predictor of impaired glucose tolerance and insulin resistance. Al-Aama et al. [21] proposed GLY and SER as predictors of early glucose metabolism disorder. Diabetic rats also showed lower GLY and SER in comparison with normal rats [22]. The authors concluded that the entry of SER to gluconeogenesis likely leads to decreased SER concentrations, suggesting that SER is one of the first amino acids affected by increased gluconeogenic activity.

The results of targeted and untargeted approaches showed a lower concentration of KYN in the MUO group compared to the MHN group. Analyzing the results of the targeted approach, the concentrations of TRP and TYR were also lower in the MUO group. A few studies have applied metabolomics approaches to investigate obesity-related metabolic disturbances in feline models. In investigating the concentration of metabolites in overweight cats undergoing weight loss, Palotto et al. [4] similarly reported lower concentration of KYN in cats with weight excess prior to weight normalization. The authors concluded that decreased TRP and KYN may indicate that synthesis of serotonin or melatonin are intensified. Hall et al. [25] investigated the metabolite profile of cats after allowing them to self-select their macronutrient intake. Cats showed intensified TRP catabolism, with higher serotonin concentrations at the expense of KYN. Also, since SER is a precursor for TRP, the contributing factor to TRP decrease could be lower SER in MUOs compared with MHNs. Contrary to our results, when investigating differences between lean and obese senior cats using a metabolomic approach, Reeve-Johnson [6] found unaltered TYR and TRP. In humans, both KYN and TRP were found to be positively associated with weight excess [26,27,28,29]. Moreover, metabolic changes in visceral adipose tissue from obese subjects were characterized by elevated levels of KYN in both metabolically healthy and unhealthy subjects [30]. The results should be interpreted in light of previous studies and the working hypotheses, addressing the broader implications of the findings and their significance. Additionally, potential directions for future research may be highlighted. 

Of the 48 metabolites that exhibited an alteration associated with excess weight, the majority were related to lipid metabolism, including triglycerides, phosphatidylcholines, lysophosphatidylcholines, and sphingomyelines. Among these, targeted metabolomics identified that 13 PCs, 2 LPCs, and 2 SMs were lower in the MUO group compared to the MHN group, while the concentrations of 19 TGs were higher. Two PCs, 39:5 and 42:7, were also lower in the MHO group compared to the MHN group. Comparing results across obesity studies is challenging due to differences in lipidomic methods, diverse study designs, and the use of various data analysis tools. Nevertheless, some results are consistent and offer at least a partial explanation of the metabolic disturbances characteristic of both animal and human obesity, suggesting a shared underlying mechanism. The same two LPCs which were found in lower concentrations in the MUO group compared to the MHNs, LPC18:1 and LPC18:2, were lower in obese children and adults [31,32,33]. In an assessment of the metabolomic changes in weight excess, using an LC-MS/MS targeted metabolomic approach, Frigeiro et al. [16] found decreased LPCs and PCs. Moreover, LPC 18:2 is suggested to be one of the most interesting negative biomarkers of obesity in humans. As a possible reason for the lower levels of LPCs and PCs in obesity, the author suggested increased lipolytic degradation due to the upregulation of specific phospholipase D. A significant negative correlation between LPC 18:2 and BCS and weight was also found in our study. Obesity-related disturbances were previously connected with lower LPC 18:2 concentrations in obese individuals compared to normal-weight ones. Investigating adiposomes, researchers found associations between LPC 18:2 and body mass index (BMI) and fat percentage [34]. Linoleate-containing lipids were found to be negatively correlated with waist circumference and body mass index in obese humans [35].

Higher TG concentration indicates increased hepatic production and secretion in MUOs. According to a hypothesis posited by Rauschert et al. [36], when fat levels exceed the storage capacity of adipocytes, fat may deposit in other tissues, where cells produce bioactive lipids that impair insulin sensitivity, leading to insulin resistance and reduced glucose uptake. Future research should investigate whether the described resistance mechanism is present in obese cats as one of the causes of the elevated glucose concentration in the MUO group. Several studies [37,38,39] have linked excess body weight, insulin resistance, and dyslipidemia, supporting the existence of a feline form of metabolic syndrome [34].

Lower levels of CIT, ADMA, SDMA, and CRE were observed in MUO cats compared to MHN cats. Of those four compounds, only CIT was found to be lower in the MHO group compared to the MHN group. Some metabolomic findings from human studies have demonstrated results similar to ours, reporting an inverse association of plasma CIT with BMI [16,40]. Also, lower CIT concentrations were found in the skeletal muscles of obese subjects compared with lean controls [41]. In addition, CIT was reduced in men with insulin resistance and T2D compared to insulin-sensitive individuals [42]. The authors concluded that CIT, as a marker of intestinal health, indicates alterations in the gut epithelium which are probably associated with obesity. In addition, the plasma concentration of CIT is considered a biomarker of enterocyte mass [42]. Lower CIT levels in both the MHO and MUO groups support the hypothesis of decreased functional enterocyte mass. Interestingly, decreased CIT was also observed in cats without metabolic alterations, raising the question of whether gut epithelial disturbances occur early in weight excess, even during the metabolically healthy stage. In our study, CIT was found to be positively correlated with the weight of the cats, contradicting the established fact that this aminoacid is reduced in obesity. This finding suggests that body weight may not be the best measure of adiposity in cats due to breed-dependent variations.

The methylated form of ARG was decreased in the MUO group compared with MHO and MHN cats. This finding is consistent with a previously reported study by Pyram et al. [43], in which the concentration of SDMA was lower in cats with diabetes mellitus than in controls. The authors suggested that this might be due to osmotic diuresis or hyperfiltration. Hillaert et al. [44], investigating the relationship between body fat and SDMA in dogs, also found a significant negative association. Since SDMA is primarily eliminated through urine [39], and given that some human obesity studies suggest obesity may involve a state of relative hyperfiltration [45,46,47], it is possible that a similar condition occurs in feline obesity, contributing to increased SDMA elimination. Our previous investigation of the impact of obesity in dogs also showed lower CRE concentrations compared to lean dogs [48]. Similarly, cats undergoing weight loss showed an increase in creatinine levels [4]. In a recent targeted metabolomics study by Frigeiro et al. [16], involving over a thousand overweight and obese subjects, CRE was negatively associated with BMI. Some of the mechanisms involved in the decrease in CRE in MUO cats could be lower muscle mass volume in relation to proliferated adipose tissue and/or a hyperfiltration state characteristic of obesity. Additionally, reduced GLY, which is required for creatine synthesis, should also be taken into account.

The highest enrichment ratio was found for thyroid hormone synthesis. Obesity, as a chronic metabolic disease, implies a positive energy balance [49]. It is well known that thyroid hormones play an important role in the regulation of energy expenditure and ability to control weight. Hypothyroidism and obesity are disorders with interrelated mechanisms—subclinical hypothyroidism can be a cause of obesity, but obesity can also affect thyroid function [50]. To evaluate the link between overweight and the future incidence of thyroid cancer in humans, some authors concluded that the rising prevalence of excess weight may contribute to approximately 10,000 thyroid cancer cases over the next decade [51].

Widely used in human medicine, metabolomics is also a valuable diagnostic tool in veterinary medicine, where it can be employed for the purpose of early diagnosis of metabolic disorders, the determination of differences in subtypes of various diseases, and as monitoring the success of therapy. Metabolomic research can identify new biomarkers for detecting disorders in metabolism even before the appearance of clinical symptoms, which enables early and more successful treatment of animals. Analyzing the complete metabolic profile in animals can improve understanding of disease mechanisms and support the development of new therapeutic strategies.

This work has two main limitations: 1. a small sample size, and 2. the use of BCS as a measure of overweight. Better insight into the concept of metabolically healthy obesity could be provided by future research on a larger number of animals with the use of a more accurate method (dual-energy X-ray absorptiometry) for assessing body fat percentage.

## 5. Conclusions

In conclusion, this study offers comprehensive metabolomic profiles of feline weight excess, identifying 48 significant metabolites through targeted metabolomics and 141 significant metabolic features through untargeted metabolomics associated with overweight in MUO cats. The major metabolites contributing to obesity were lipid metabolites. Four metabolites were also differentially abundant in the MHO group, supporting the hypothesis that cats with a healthy phenotype exhibit an intermediate-stage metabolic risk profile. These weight-related metabolite changes challenge the concept of metabolically healthy overweight and obesity in cats. Many identified metabolites have been previously linked to impaired glucose and energy metabolism and the potential development of insulin resistance. Therefore, it would be valuable to investigate, in a larger cohort over a longer period, whether these altered metabolites contribute to the development of feline-obesity-related metabolic diseases. In summary, this study revealed the impact of feline weight excess on the metabolome using targeted and untargeted metabolomics, advancing our understanding of obesity-related metabolic disturbances and aiding in identifying cats at higher risk of obesity-related diseases.

## Figures and Tables

**Table 1:** Biochemical characteristics and body condition score of the study population of cats.

		MHN			MHO			MUO		
Parameter	Unit	MEAN	MIN	MAX	MEAN	MIN	MAX	MEAN	MIN	MAX
BCS		5	5	5	7.0	6	9	7.0	6	8
BUN	mmol/L	7.8	3.9	11	6.9	4.5	8.6	9.7	7.2	15
CRE	µmol/L	97	74	142	100	62	188	98	72	153
PRO	g/L	73	64	85	73	57	87	81	72	88
ALB	g/L	27	23	30	27	24	30	28	25	31
BIL	µmol/L	1.3	0.9	1.7	1.9	0.9	4.9	1.8	0.9	4.4
GLU	mmol/L	5.3	4.2	6.6	4.8	1.9	6.4	13	7.7	25
AST	IU/L, 37 °C	19	10	37	21	9.0	42	30	8.0	98
ALT	IU/L, 37 °C	56	34	149	61	36	112	78	40	153
AP	IU/L, 37 °C	65	24	135	48	20	104	42	22	60
TG	mmol/L	0.6	0.2	1.1	0.6	0.2	1.2	1.9	0.6	5.4
CHOL	mmol/L	2.9	2.0	4.0	3.0	2.2	3.8	5.9	4.8	8.8
ADP	µg/mL	4.2	1.0	7.5	2.4	1.3	4.6	1.5	0.7	2.5

MHNs—metabolically healthy normal-weight cats, MHOs—metabolically healthy overweight and obese cats, MUOs—metabolically unhealthy overweight and obese cats, BCS—body condition score, BUN—blood urea nitrogen, CRE—creatinine, PRO—total protein, ALB—albumin, BIL—total bilirubin, GLU—glucose, AST—aspartate aminotransferase, ALT—alanine aminotransferase, AP—alkaline phosphatase, TGs—triglycerides, CHOL—cholesterol, ADP—adiponectin.

**Table 2:** List of identified and significantly changed metabolites with differential abundance between MHOs, MUOs, and MHLs as determined by the targeted LC-MS and FIA-MS approach.

Metabolite	*p*	FDR	Post Hoc Significance		
Kynurenine	2.90 × 10^−6^	0.000577	MHN/MUO	MHO/MUO	
Gly	9.84 × 10^−6^	0.000979	MHN/MHO	MHN/MUO	MHO/MUO
TG (52:6)	1.73 × 10^−5^	0.000985	MUO/MHN	MUO/MHO	MUO/MHO
SM (40:4)	2.34 × 10^−5^	0.000985	MHN/MUO	MHO/MUO	
TG (53:3)	2.48 × 10^−5^	0.000985	MUO/MHN	MUO/MHO	
PC (42:3)	5.10 × 10^−5^	0.001615	MHN/MUO	MHO/MUO	
Ser	5.68 × 10^−5^	0.001615	MHN/MUO	MHO/MUO	
TG (54:7)	0.000154	0.00384	MUO/MHN	MUO/MHO	
SM (31:1)	0.00021	0.00465	MHN/MUO	MHO/MUO	
TG (54:6)	0.00029	0.005767	MUO/MHN	MUO/MHO	
ADMA	0.000375	0.006493	MHN/MUO	MHO/MUO	
TG (52:4)	0.000392	0.006493	MUO/MHN	MUO/MHO	
TG (56:6)	0.000481	0.007365	MUO/MHN	MUO/MHO	
Tyr	0.00054	0.007673	MHN/MUO	MHO/MUO	
TG (51:3)	0.000602	0.007988	MUO/MHN	MUO/MHO	
TG (56:7)	0.000676	0.008048	MUO/MHN	MUO/MHO	
Pro	0.000687	0.008048	MHN/MUO	MHO/MUO	
PC (33:0)	0.000896	0.009132	MHN/MUO	MHO/MUO	
Cit	0.000913	0.009132	MHN/MHO	MHN/MUO	
PC (39:5)	0.000918	0.009132	MHN/MHO	MHN/MUO	MHO/MUO
TG (52:5)	0.001179	0.011175	MUO/MHN	MUO/MHO	
PC-O (40:7)	0.001288	0.011648	MHN/MUO	MHO/MUO	
PC (37:5)	0.001403	0.011905	MHN/MUO	MHO/MUO	
TG (54:5)	0.001472	0.011905	MUO/MHN	MUO/MHO	
LPC (18:1)	0.001496	0.011905	MHN/MUO	MHO/MUO	
TG (53:4)	0.002004	0.014842	MUO/MHN	MUO/MHO	
PC (34:5)	0.002032	0.014842	MHN/MUO	MHO/MUO	
Cer (34:0)	0.002088	0.014842	MHN/MUO	MHO/MUO	
TG (52:2)	0.002237	0.01535	MUO/MHN	MUO/MHO	
TG (50:4)	0.002529	0.016257	MUO/MHN	MUO/MHO	
PC (24:0)	0.002555	0.016257	MHN/MUO	MHO/MUO	
TG (51:2)	0.002614	0.016257	MUO/MHN	MUO/MHO	
TG (52:3)	0.002812	0.016958	MUO/MHN	MUO/MHO	
Creatinine	0.003092	0.018095	MHN/MUO	MHO/MUO	
Trp	0.003213	0.018265	MHN/MUO	MHO/MUO	
LPC (18:2)	0.003337	0.018406	MHN/MUO	MHO/MUO	
SDMA	0.003509	0.018406	MHN/MUO	MHO/MUO	
PC-O (26:1)	0.003515	0.018406	MHN/MUO	MHO/MUO	
PC (37:7)	0.003745	0.018724	MHN/MUO	MHO/MUO	
PC (44:10)	0.003764	0.018724	MHN/MUO	MHO/MUO	
PC (36:5)	0.00447	0.021252	MHN/MUO	MHO/MUO	
Putrescine	0.004485	0.021252	MHN/MUO	MHO/MUO	
TG (54:4)	0.004875	0.022559	MUO/MHN	MUO/MHO	
TG (50:3)	0.005183	0.02344	MUO/MHN	MUO/MHO	
TG (56:8)	0.007278	0.032184	MUO/MHN	MUO/MHO	
PC (37:2)	0.008218	0.035549	MHN/MUO		
PC (42:7)	0.009626	0.040756	MHN/MHO	MHN/MUO	
TG (54:3)	0.011436	0.047411	MUO/MHN	MUO/MHO	

MHNs—metabolically healthy normal-weight cats, MHOs—metabolically healthy overweight and obese cats, MUOs—metabolically unhealthy overweight and obese cats. Gly—glycin, TGs—triglycerides, SMs—sphingomyelins, PCs—phosphatidylcholines, SER—serine, ADMA—asimmetric dimethylarginine, TYR—tyrosine, PRO—proline, CIT—citruline, LPCs—lysophosphatidylcholines, CER—ceramide, TRP—tryptophane, SDMA—asimmetric dimethylarginine.

## Data Availability

The metabolomics and metadata reported in this paper are available via MetaboLights (https://www.ebi.ac.uk/metabolights/MTBLS8725, accessed on 23 July 2025).

## Supplementary Materials

The following supporting information can be downloaded at: https://www.mdpi.com/article/10.3390/vetsci12080697/s1, Table S1: Phenotypic characteristics of cats included in the study.
